# Comparison of hospital variation in acute myocardial infarction care and outcome between Sweden and United Kingdom: population based cohort study using nationwide clinical registries

**DOI:** 10.1136/bmj.h3913

**Published:** 2015-08-07

**Authors:** Sheng-Chia Chung, Johan Sundström, Chris P Gale, Stefan James, John Deanfield, Lars Wallentin, Adam Timmis, Tomas Jernberg, Harry Hemingway

**Affiliations:** 1 Farr Institute of Health Informatics Research and Institute of Health Informatics, University College London, London NW1 2DA, UK; 2 Department of Medical Sciences, Cardiology, Uppsala University, Uppsala, Sweden; 3 Uppsala Clinical Research Center, Uppsala, Sweden; 4 Cardiovascular Health Sciences, University of Leeds, Leeds, UK; 5 National Centre for Cardiovascular Prevention and Outcomes, London, UK; 6 National Institute for Health Research, Cardiovascular Biomedical Research Unit, Bart’s Health London, London, UK; 7 Department of Medicine, Huddinge, Section of Cardiology, Karolinska Institute, Stockholm, Sweden

## Abstract

**Objective** To assess the between hospital variation in use of guideline recommended treatments and clinical outcomes for acute myocardial infarction in Sweden and the United Kingdom.

**Design** Population based longitudinal cohort study using nationwide clinical registries.

**Setting and participants** Nationwide registry data comprising all hospitals providing acute myocardial infarction care in Sweden (SWEDEHEART/RIKS-HIA, n=87; 119 786 patients) and the UK (NICOR/MINAP, n=242; 391 077 patients), 2004-10.

**Main outcome measures** Between hospital variation in 30 day mortality of patients admitted with acute myocardial infarction.

**Results** Case mix standardised 30 day mortality from acute myocardial infarction was lower in Swedish hospitals (8.4%) than in UK hospitals (9.7%), with less variation between hospitals (interquartile range 2.6% *v* 3.5%). In both countries, hospital level variation and 30 day mortality were inversely associated with provision of guideline recommended care. Compared with the highest quarter, hospitals in the lowest quarter for use of primary percutaneous coronary intervention had higher volume weighted 30 day mortality for ST elevation myocardial infarction (10.7% *v* 6.6% in Sweden; 12.7% *v* 5.8% in the UK). The adjusted odds ratio comparing the highest with the lowest quarters for hospitals’ use of primary percutaneous coronary intervention was 0.70 (95% confidence interval 0.62 to 0.79) in Sweden and 0.68 (0.60 to 0.76) in the UK. Differences in risk between hospital quarters of treatment for non-ST elevation myocardial infarction and secondary prevention drugs for all discharged acute myocardial infarction patients were smaller than for reperfusion treatment in both countries.

**Conclusion** Between hospital variation in 30 day mortality for acute myocardial infarction was greater in the UK than in Sweden. This was associated with, and may be partly accounted for by, the higher practice variation in acute myocardial infarction guideline recommended treatment in the UK hospitals. High quality healthcare across all hospitals, especially in the UK, with better use of guideline recommended treatment, may not only reduce unacceptable practice variation but also deliver improved clinical outcomes for patients with acute myocardial infarction.

**Clinical trials registration** Clinical trials NCT01359033.

## Introduction

Variation between hospitals in quality of care and clinical outcomes provides useful insight into the performance of national health systems.^1^ ^2^ ^3^ ^4^ ^5^ ^6^ ^7^ ^8^ Considerable attention has focused on acute myocardial infarction,^3^ ^4^ ^5^ ^6^ ^7^ ^8^ ^9^ ^10^ which remains the leading cause of cardiovascular death worldwide. Within country studies have reported declining between hospital variation in mortality from acute myocardial infarction over recent years,^3^ ^6^ ^11^ but they have been inconsistent in attributing this to reductions in variation in practice.^4^ ^5^ Most studies have reported practice variation and outcomes independently and have been limited by lack of population coverage, basing their analyses on selected hospitals or on subgroups defined by age or acute myocardial infarction phenotype (either ST elevation myocardial infarction or non-ST elevation myocardial infarction).^3^ ^4^ ^5^ ^9^ ^10^

To date, analyses of between hospital variation in the treatment and outcomes of acute myocardial infarction have been conducted within countries; although this has provided information about national scope for improvement in health systems, it has been uninformative about systems’ performance relative to other countries. This represents an important missed opportunity for learning from other healthcare systems and for international benchmarking of performance. Here, we report the variation between hospitals in the treatment and outcomes of patients with acute myocardial infarction in Sweden and the United Kingdom (England and Wales), the only two countries in the world with continuous national quality of care and outcome registries for acute myocardial infarction in which all hospitals participated.^12^ ^13^ The comparison was facilitated and made more relevant by the similarity of the health systems in the two countries, both of which provide tax funded universal care free at the point of contact.

We have previously reported a significant difference in patients’ 30 day mortality after acute myocardial infarction between Sweden and the UK,^14^ and the difference in mortality reduced with time. We have now sought to determine whether between hospital variation in acute myocardial infarction care and clinical outcomes also differ between the two countries. Our specific objectives were to compare Sweden and the UK in terms of between hospital variation in the use of guideline recommended treatment for acute myocardial infarction and the crude and case mix standardised 30 day mortality. We investigated possible time trends, sources of between hospital variation, and potential benefits of targeting between hospital variation for better adherence to guideline recommended treatment to improve survival of patients with acute myocardial infarction.

## Methods

### Eligible hospitals and patients

We included all acute hospital units providing care for patients with acute myocardial infarction in Sweden (n=87) and in England and Wales (n=242). Eligible patients (119 786 patients in Sweden and 391 077 patients in the UK) were aged at least 30 years and admitted between 1 January 2004 and 31 December 2010. The diagnosis of acute myocardial infarction was based on guidelines from the European Society of Cardiology, American College of Cardiology, and American Heart Association.^15^ For multiple admissions of the same patient, we used the earliest record. Details of data validation,^12^ ^13^ patient population, data quality and completeness, and comparability of variable definitions regarding acute myocardial infarction diagnosis, patient case mix, evidence based hospital treatment strategies, and discharge drug variables in SWEDEHEART/RIKS-HIA and MINAP were described in the previous study.^14^

### Case mix variables

The 17 case mix measures were demographic factors (age, sex, year of hospital admission); risk factors (smoking, history of diabetes and hypertension); severity of acute myocardial infarction (troponin I or T concentration, systolic blood pressure and heart rate at admission to hospital, and cardiac arrest); history of previous heart failure, cerebrovascular disease, and acute myocardial infarction; and procedure and drug use before admission (antiplatelet treatment with aspirin, clopidogrel, or both; previous percutaneous coronary intervention and coronary artery bypass graft).

### Guideline recommended treatment

Class 1 guideline recommended treatment included reperfusion treatment (primary percutaneous coronary intervention, any fibrinolytic treatment) for patients with ST elevation myocardial infarction; revascularisation (percutaneous coronary intervention or coronary artery bypass graft) as appropriate and feasible, and anticoagulant (unfractionated heparin, low molecular weight heparin, or Fondaparinux) for patients with non-ST elevation myocardial infarction; and, for patients with acute myocardial infarction who survived to hospital discharge, the use of antiplatelet treatment (single or dual antiplatelet), β blockers, angiotensin converting enzyme inhibitors or angiotensin receptor blockers, and 3-hydroxy-3-methylglutaryl coenzyme A reductase inhibitors (statins).^16^ ^17^ ^18^ ^19^

### Mortality outcomes

The primary clinical outcome was all cause mortality within 30 days of hospital admission. Each country has a national unique identifier which we used to link patients with the National Population Registry (in Sweden) and the Office for National Statistics (in the UK) to ascertain vital status and date of death (available for 99.3% and 98.7% of patients, respectively).

### Statistical analysis

We compared the crude hospital proportions of case mix, treatments, and 30 day mortality by year of hospital admission. We initially investigated the hospital trajectories of these proportions graphically by using panel plots. We derived hospitals’ use of treatments and 30 day mortality rates from patient data and weighted them by each hospital’s total volume of patients with acute myocardial infarction, overall and by admission year.

As the hospital rates were not normally distributed, we used the interquartile range to describe the observed variation. To account for patient case mix in investigating variation in hospital practice, we constructed case mix models for each of the guideline recommended treatments and 30 day mortality by using the 17 variables described above. Using these models, we estimated the predicted probability of receipt of guideline recommended treatments and death at 30 days after hospital admission for each patient and summarised these at the hospital level to obtain the expected hospital proportions. For each hospital, we divided the observed mortality by the expected hospital 30 day mortality to obtain the hospital standardised mortality ratio. We determined hospitals’ case mix standardised 30 day mortality rates by multiplying each hospital’s standardised mortality ratio by the crude 30 day mortality rate for acute myocardial infarction for the respective country. Likewise, we applied the indirect standardisation to obtain case mix standardised hospital treatment proportions. We estimated the association between case mix standardised hospital treatment proportions and standardised hospital mortality by correlation analysis.

To examine the association between hospitals’ variation in treatments and patients’ risk of 30 day mortality, we first summarised volume weighted hospital 30 day mortality rates by quarters of hospital guideline recommended care, in which we classified hospitals by proportions of treatment use into lowest (first quarter), medium-low (second quarter), medium-high (third quarter), and highest (fourth quarter). We fitted a multilevel generalised mixed model, adding the variables of hospital treatment quarters (hospital level variables) to the 17 case mix variables (patient level variables), to compare the case mix adjusted risk ratios between quarters of hospital treatment use and mortality risk in Sweden and the UK. The model incorporated a hospital random effect to account for clustering of patient data. We evaluated the extent of patients with missing data for the case mix variables and managed it by multiple imputation (supplementary appendix 1). We examined the model fit by the distribution of Pearson residuals and mean square weighted deviation (Pearson χ^2^ statistic divided by degree of freedom) (supplementary appendix 2).^20^ We did analyses separately for each country with a common protocol, using SAS version 9.0 in London by means of secure remote access.

### Patient involvement

No patients were involved in setting the research question or the outcome measures, and nor were they involved in the design and implementation of the study. We plan to involve patients in dissemination of the study results by inviting patients and their families to the study information session hosted at the Farr Institute.

## Results

### Hospital level case mix

Hospital level measures of infarct severity, including heart rate, systolic blood pressure, and troponin concentration, were comparable for Sweden and the UK (supplementary table A). Compared with hospitals in the UK, we observed a higher median proportion of female patients (37.0% *v* 34.6%) and patients with a history of diabetes (22.8% *v* 17.0%) or heart failure (9.5% *v* 5.1%) in Swedish hospitals. Although the proportion of patients with ST elevation myocardial infarction was greater in UK hospitals than in Swedish hospitals between 2004 and 2007, it was more comparable in 2010 (27.5% (interquartile range 30.5%) *v* 31.3% (11.2%), respectively). Hospital volume was greater in Sweden than in the UK, with a median of 2325 in 2004 and 2349 in 2010, compared with 1982 and 2084 in the UK.

### Hospital level treatment

Hospital provision of primary percutaneous coronary intervention for ST elevation myocardial infarction patients was higher in Sweden (61.9% *v* 34.9%), and variation was much lower (interquartile range 16.7% *v* 50.7%), compared with the UK. Figure 1 shows that in both countries, reduced between hospital variation in the provision of primary percutaneous coronary intervention coexisted with its temporal increase between 2004 and 2010 (interquartile range for Sweden 41.6% to 12.8% versus 29.2% to 17.3% for UK). Of note, the use of any reperfusion treatment for ST elevation myocardial infarction was greater in UK hospitals over the study period (and predominantly driven by the greater use of thrombolytic treatment); whereas between hospital variation in its use decreased in Sweden, it increased in the UK. Hospital provision of revascularisation for non-ST elevation myocardial infarction patients was lower in the UK than in Sweden (19.2% *v* 34.8%), with twice the size of variation (interquartile range 21.9% in the UK and 10.2% in Sweden). Hospital use and practice variation in provision of anticoagulant for non-ST elevation myocardial infarction patients were similar in Sweden and the UK.

In the pre-discharge prescription of secondary prevention drugs recommended by guidelines, between hospital variation was lower for antiplatelet drugs than for other secondary prevention drugs in both Sweden (interquartile range 4.7% in 2004 and 2.9% in 2010) and the UK (4.8% in 2004 and 4.2% in 2010) (fig 2). The prescription of β blockers was higher in Sweden than in the UK, with less variation in Swedish hospitals (interquartile range 4.9%) than in UK hospitals (10.1%). Conversely, the prescription of angiotensin converting enzyme inhibitors/angiotensin receptor blockers and statins was higher in UK hospitals than in Swedish hospitals, with somewhat greater variation in Sweden (11.6% and 11.9%, respectively) than in the UK (10.1% and 4.8%).

**Figure fig1:**
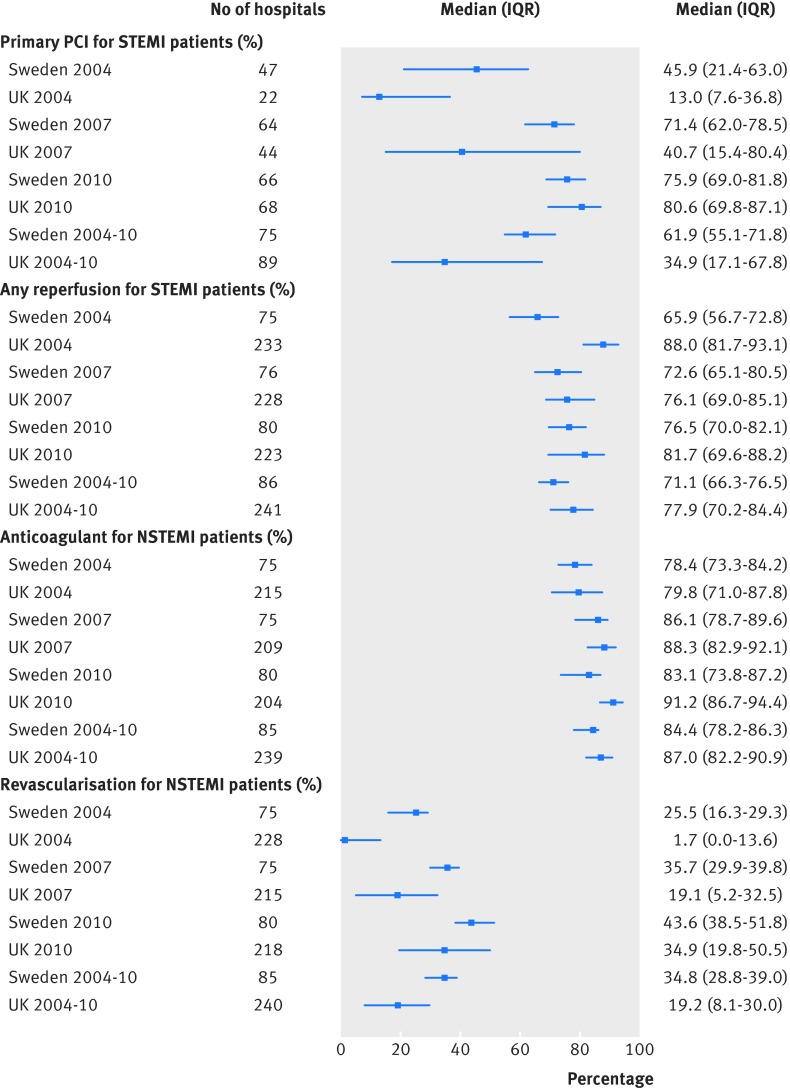
**Fig 1** Hospital variation in use (median percentage and interquartile range) of treatment for ST elevation myocardial infarction (STEMI) and non- ST elevation myocardial infarction (NSTEMI) by year in Sweden and UK. Reperfusion percentage among STEMI patients was weighted by number of STEMI admissions to hospital. Primary percutaneous coronary intervention (PCI) rate was among hospitals that did ≥5 primary PCIs during year. Percentages of revascularisation and anticoagulant were weighted by NSTEMI admissions. Anticoagulant included heparin

**Figure fig2:**
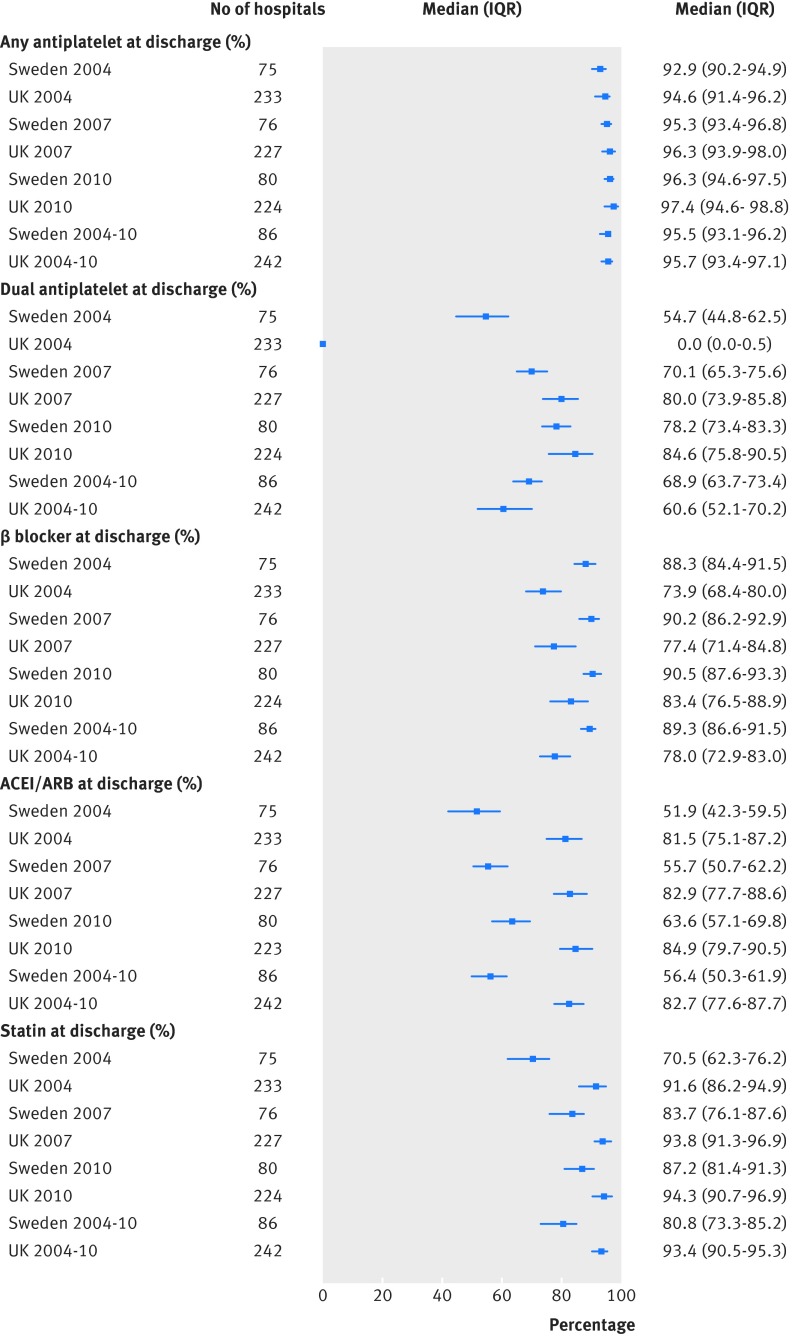
**Fig 2** Hospital variation in use (median percentage and interquartile range) of discharge drugs for acute myocardial infarction (AMI) by year in Sweden and UK. Restricted to patients who survived beyond discharge and weighted by hospital AMI volume. ACEI=angiotensin converting enzyme inhibitor; ARB=angiotensin receptor blocker

### Inter-hospital variation in 30 day mortality

During the study period, volume weighted 30 day mortality in Swedish hospitals (7.0%) was lower than in UK hospitals (10.1%) and showed less variation (interquartile range 2.1% *v* 5.7%) (fig 3). Thirty day mortality and its variation decreased between 2004 and 2010 from 9.3% (interquartile range 3%) to 6.5% (2.8%) in Swedish hospitals and from 12.8% (7.5%) to 7.6% (5.5%) in UK hospitals (fig 3). In Sweden, between hospital variation in 30 day mortality was greater for ST elevation myocardial infarction (interquartile range 2.5%) than for non-ST elevation myocardial infarction (1.8%), in contrast to the UK where the variation was greater for non-ST elevation myocardial infarction (7.1%) than for ST elevation myocardial infarction (5.4%). After control for the difference in case mix of admitted patients, the hospital case mix standardised 30 day mortality and its variance remained lower in Sweden at 8.4% (interquartile range 2.6%) compared with 9.7% (3.5%) in the UK (fig 4).

**Figure fig3:**
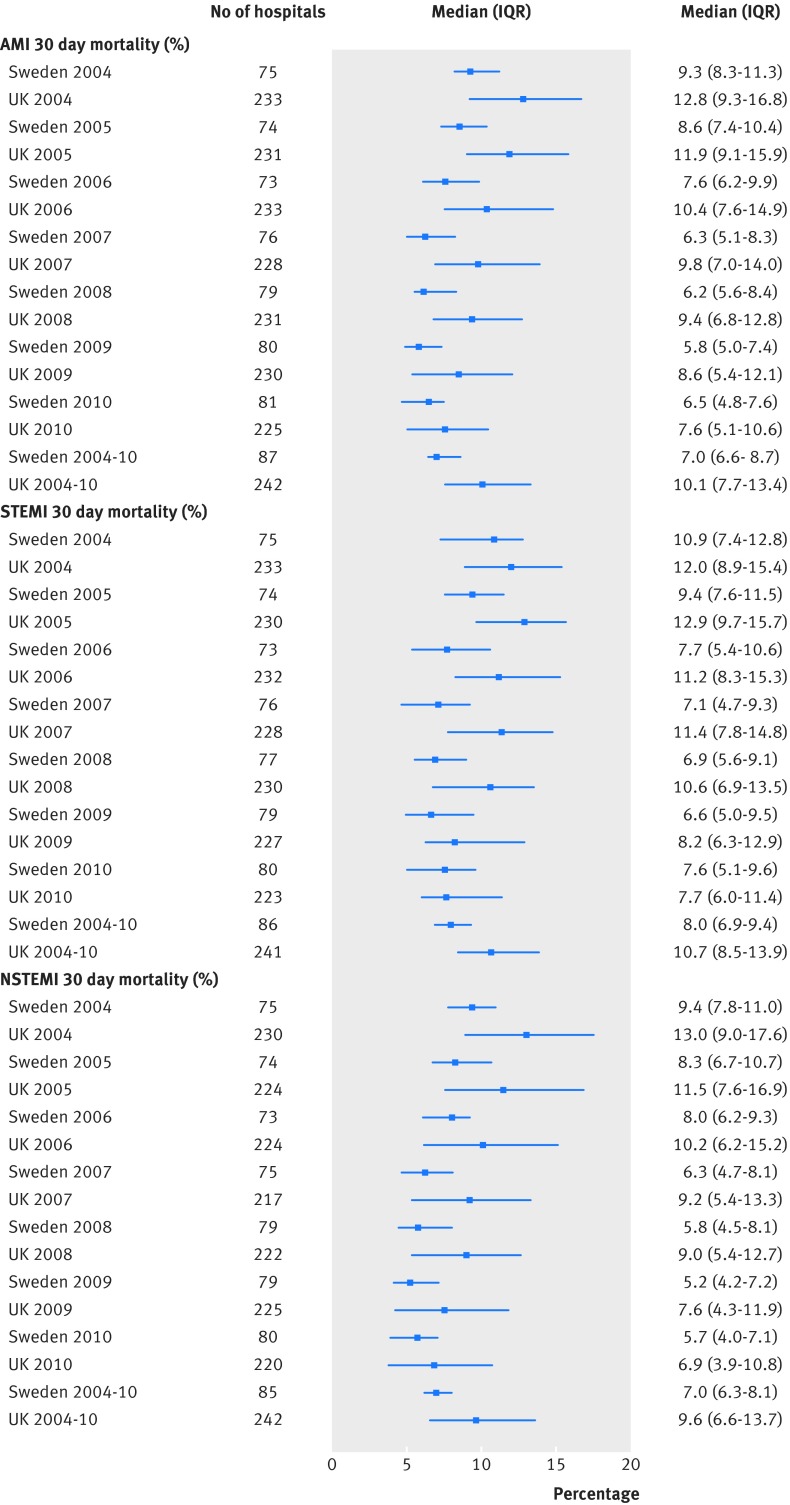
**Fig 3** Variation in acute myocardial infarction (AMI), ST elevation myocardial infarction (STEMI), and non-ST elevation myocardial infarction (NSTEMI) 30 day mortality (median percentage and interquartile range) by year in Swedish and UK hospitals. Weighted by hospital volume of AMI, STEMI, and NSTEMI, respectively

**Figure fig4:**
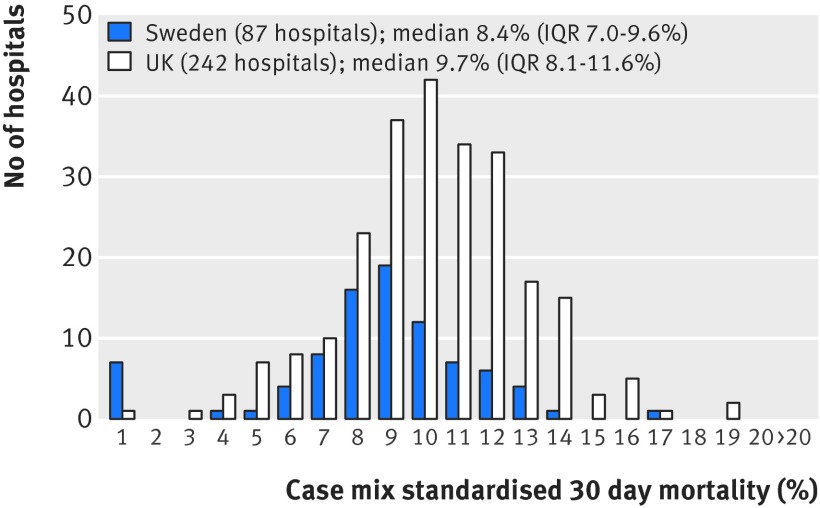
**Fig 4** Hospital variation in case mix standardised 30 day mortality (%) in Sweden and UK, 2004-10

### Association between hospital variation in treatment and in AMI 30-day mortality

In Sweden, after control for difference in case mix of admitted patients, variation in hospital provision of guideline recommended treatment together explained 28.1% of the between hospital variation in 30 day mortality from acute myocardial infarction; variation in reperfusion therapy for ST elevation myocardial infarction and statins prescribed at discharge probably played an important role. Variation in hospital treatment explained 21.6% of the variation in 30 day mortality from acute myocardial infarction in UK hospitals, where provision of primary percutaneous coronary intervention, antiplatelet treatment, and statins seemed to be important (supplementary table B).

### Acute myocardial infarction 30 day mortality by hospital treatment quarters

In both Sweden and the UK, lower volume weighted 30 day mortality rates occurred in hospitals in the highest quarter for use of guideline recommended treatments (table[Table tbl1]). The difference was most notable for primary percutaneous coronary intervention, for which comparison of hospitals in the lowest and highest quarters showed volume weighted 30 day mortality rates of 10.7% (95% confidence interval 10.1% to 11.3%) versus 6.6% (6.0% to 7.1%) in Sweden and of 12.7% (12.4% to 13.0%) versus 5.8% (5.1% to 6.4%) in the UK. The difference in mortality by quarters of hospital treatment decreased after standardisation by patient case mix (table of supplementary appendix 3). After adjustment for patient case mix, compared with ST elevation myocardial infarction patients admitted to hospitals in the lowest quarter, those in hospitals in the highest quarter had a 30% lower risk of death at 30 days in Sweden and a 32% lower risk in the UK (adjusted odds ratio of 0.70 (95% confidence interval 0.62 to 0.79) in Sweden and 0.68 (0.60 to 0.76) in the UK) (fig 5). The difference in odds ratio was smaller for the comparison of quarter 1 (lowest) with quarter 2 or 3 hospitals. We also observed a smaller difference in odds ratios between hospital quarters in any reperfusion for ST elevation myocardial infarction and in revascularisation and anticoagulant use for non-ST elevation myocardial infarction patients. By admission year, a similar trend existed for diminishing risk of 30 day mortality for ST elevation myocardial infarction patients as the hospital provision of primary percutaneous coronary intervention or any reperfusion treatment increased, especially in the UK (supplementary figure A). For discharge drugs, the case mix adjusted odds ratios comparing hospitals in the highest and lowest quarters for Sweden and the UK were, respectively, 0.92 (0.84 to 1.01) and 0.87 (0.83 to 0.92) for any antiplatelet drug, 0.83 (0.74 to 0.92) and 0.88 (0.83 to 0.94) for dual antiplatelet treatment, 1.04 (0.95 to 1.13) and 0.84 (0.80 to 0.88) for β blockers, 0.87 (0.79 to 0.95) and 0.89 (0.85 to 0.94) for angiotensin converting enzyme inhibitors/angiotensin receptor blockers, and 0.84 (0.76 to 0.93) and 0.84 (0.81 to 0.88) for statins (fig 5).

In the hypothetical scenario, if hospitals in the lower three quarters for primary percutaneous coronary intervention use had the average case mix standardised mortality in the highest quarter, the estimated reduction in deaths would be 581 in Sweden and 1013 in the UK. Likewise, the reduction in deaths would be 573 in Sweden and 2274 in the UK if hospitals with lower use of dual antiplatelet treatment at discharge reached the case mix standardised mortality of hospitals with the highest use (supplementary appendix 3).

**Figure fig5:**
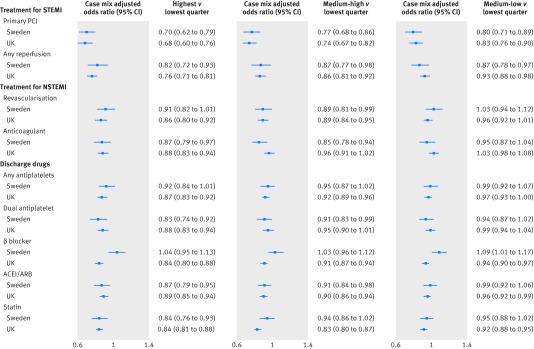
**Fig 5** Estimated case mix adjusted 30 day mortality for ST elevation myocardial infarction (STEMI), non-ST elevation myocardial infarction (NSTEMI), and acute myocardial infarction (AMI) patients and odds ratio by hospital treatment quarters, in Sweden and UK. STEMI mortality reported by hospital reperfusion treatment quarters; NSTEMI mortality reported by hospital revascularisation and anticoagulant use quarters; AMI mortality reported by hospital discharge drug use quarters. ACEI=angiotensin converting enzyme inhibitor; ARB=angiotensin receptor blocker

**Table tbl1:** Estimated case mix adjusted 30 day mortality by quarters of hospital treatment

Country	No of hospitals	Volume weighted mortality (95% CI) by hospital treatment quarters			
		Highest	Medium-high	Medium-low	Lowest
Treatment for ST elevation myocardial infarction*					
Primary PCI:					
Sweden	86	6.6 (6.0 to 7.1)	7.6 (7.0 to 8.2)	8.2 (7.6 to 8.9)	10.7 (10.1 to 11.3)
UK	241	5.8 (5.1 to 6.4)	8.2 (7.3 to 9.1)	8.8 (8.0 to 9.6)	12.7 (12.4 to 13.0)
Any reperfusion:					
Sweden	86	7.1 (6.5 to 7.6)	7.7 (7.0 to 8.4)	8.5 (7.8 to 9.2)	10.5 (9.8 to 11.1)
UK	241	8.0 (7.6 to 8.4)	10.5 (10.0 to 10.9)	12.1 (11.6 to 12.6)	14.4 (13.9 to 15.0)
**Treatment for non-ST elevation myocardial infarction†**					
Revascularisation:					
Sweden	85	6.1 (5.7 to 6.5)	6.3 (5.8 to 6.7)	8.1 (7.6 to 8.6)	8.6 (8.0 to 9.2)
UK	240	7.9 (7.4 to 8.5)	9.8 (9.3 to 10.3)	11.7 (11.1 to 12.3)	11.2 (10.6 to 11.8)
Anticoagulant:					
Sweden	84	6.8 (6.4 to 7.3)	6.5 (6.1 to 7.0)	7.9 (7.3 to 8.4)	7.8 (7.3 to 8.4)
UK	239	7.9 (7.5 to 8.3)	9.8 (9.3 to 10.4)	11.2 (10.5 to 11.8)	12.6 (12.0 to 13.3)
**Discharge drugs for acute myocardial infarction‡**					
Any antiplatelets:					
Sweden	86	6.2 (5.9 to 6.5)	7.0 (6.6 to 7.4)	7.9 (7.4 to 8.3)	9.5 (9.0 to 10.0)
UK	242	7.9 (7.5 to 8.3)	9.7 (9.2 to 10.1)	11.6 (11.1 to 12.0)	13.2 (12.7 to 13.7)
Dual antiplatelet:					
Sweden	86	6.1 (5.7 to 6.4)	6.8 (6.4 to 7.2)	7.9 (7.4 to 8.3)	9.8 (9.3 to 10.3)
UK	242	7.7 (7.3 to 8.1)	10.2 (9.7 to 10.7)	11.5 (11.1 to 12.0)	12.9 (12.4 to 13.4)
β blocker:					
Sweden	86	7.1 (6.7 to 7.6)	7.9 (7.3 to 8.4)	7.8 (7.3 to 8.3)	7.8 (7.4 to 8.2)
UK	242	8.1 (7.7 to 8.5)	9.6 (9.1 to 10.0)	11.5 (11.1 to 12.0)	13.2 (12.7 to 13.7)
ACEI/ARB:					
Sweden	86	6.4 (6.0 to 6.8)	7.0 (6.6 to 7.4)	8.3 (7.8 to 8.8)	9.0 (8.5 to 9.4)
UK	242	8.9 (8.4 to 9.3)	10.4 (9.9 to 10.8)	11.1 (10.6 to 11.6)	12.1 (11.6 to 12.6)
Statin:					
Sweden	86	6.0 (5.6 to 6.3)	7.2 (6.8 to 7.5)	7.7 (7.3 to 8.1)	9.7 (9.2 to 10.2)
UK	242	8.4 (8.0 to 8.8)	9.3 (8.8 to 9.8)	10.9 (10.5 to 11.3)	13.7 (13.2 to 14.2)

ACEI=angiotensin converting enzyme inhibitor; ARB=angiotensin receptor blocker; PCI=percutaneous coronary intervention

*Mortality reported by quarters of hospital reperfusion treatment.

†Mortality reported by quarters of hospital revascularisation and anticoagulant use.

‡Mortality reported by quarters of hospital discharge drug use.

## Discussion

This is the first hospital level international comparison of the characteristics, treatments, and clinical outcomes of patients with acute myocardial infarction admitted to all hospitals across Sweden and the United Kingdom. In both countries, greater use of guideline recommended treatment in hospitals was associated with smaller variation in practice. Hospitals’ practice variation and 30 day mortality were inversely related. Patients’ risk of 30 day mortality was lower in hospitals in the upper than the lower quarters of treatment use, after adjustment for case mix. Comparative analysis showed that, in both countries, variation between hospitals in the use of primary percutaneous coronary intervention, antiplatelet treatment, and statin at discharge were important in explaining variation in 30 day mortality. Unmeasured factors accounted for more variation in mortality from acute myocardial infarction in UK hospitals than in Swedish hospitals. The data suggest that more consistent adherence to new treatment guidelines across all hospitals would not only reduce practice variation but also deliver improved outcomes.

### Treatment variation

Admission rates for acute myocardial infarction were greater in Sweden than in the UK.^21^ ^22^ ^23^ This finding was similar to the greater rate of ischaemic heart disease reported in Sweden than the UK on the basis of discharge data,^24^ suggesting a higher rate of coronary heart disease in hospitals in Sweden than in the UK. Hospitals in the UK showed a distinct temporal trend in the use of reperfusion treatment; the clinical consensus seemed to favour thrombolytic treatment from 2004 to 2007, with increasing use of primary percutaneous coronary intervention thereafter but not matching Sweden’s use until 2010. The switch towards increasing use of primary percutaneous coronary intervention in UK hospitals in 2008 was associated with a significant reduction in inter-hospital variation in practice and corresponded with the launch of a national policy initiative the same year favouring primary percutaneous coronary intervention.^25^ ^26^ In Sweden, reductions in variation in the use of primary percutaneous coronary intervention between hospitals diminished in 2006, reflecting a national consensus that had moved away from thrombolytic treatment at least two years earlier than in the UK. This two year delay in implementation of primary percutaneous coronary intervention in the UK seems to have had adverse consequences, judging by the favourable 30 day odds of mortality for patients with ST elevation myocardial infarction receiving treatment in high use compared with low use hospitals. Our data suggest that reducing between hospital variation in primary percutaneous coronary intervention by increasing its use among hospitals with lower adherence has the potential to reduce the mortality risk of ST elevation myocardial infarction in both countries.

Between hospital variation in the use of guideline recommended treatment for non-ST elevation myocardial infarction patients and secondary prevention therapy was less marked than for reperfusion treatment in Sweden and the UK. The lowest variation in hospital practice was observed in the use of any antiplatelet drug, with a between hospital variation of less than 4% in both countries, which may be associated with the close to complete hospital provision. The adjusted odds ratios for patients’ 30 day mortality by quarters of hospitals’ use of any antiplatelet treatment were close to null. The low levels of inter-hospital variation in any antiplatelet drug treatment with little difference in mortality between hospitals may provide a useful benchmark for other secondary prevention drugs. If variations between Swedish and UK hospitals in prescription of all secondary prevention drugs were reduced to the levels recorded for any platelet drugs, important reductions in variation in mortality could probably be achieved.

### Mortality variation

A major finding of this study was that hospital level 30 day mortality for patients with acute myocardial infarction was not only higher in the UK but also showed greater variation than in Sweden. Thus, the distribution of case mix standardised 30 day mortality for UK hospitals in figure 4 was broader based and shifted to the right compared with Swedish hospitals. Our data suggest that variations in hospital treatment explained about 28% of variation in mortality in Swedish hospitals and 22% in UK hospitals, which was significantly greater than the 6% reported by a previous population study.^4^ Although risk differences between hospital quarters for secondary prevention drugs were smaller than for reperfusion treatment, the potential for deaths prevented or deferred is similar or greater for reducing variation in use of secondary prevention drugs, which benefits all patients with myocardial infarction, than for reducing variation in reperfusion treatment, which benefits only those with ST elevation myocardial infarctions (supplementary appendix 3).

### Policy implications

Other investigators have reported that, beyond variations in hospital treatment, multiple factors might contribute to the residual variation in mortality between hospitals, including hospital structure (staff expertise, hospital volume, resources),^8^ ^10^ ^27^ ^28^ ^29^ ^30^ ^31^ ^32^ processes of care (treatment protocol, problem solving),^8^ ^27^ ^29^ ^31^ ^33^ and organisational culture.^8^ ^29^ ^31^ ^33^ We found that more patients in both Sweden and the UK were admitted to hospitals in the lowest quarters of treatment use than to those in the higher quarters. Although volume weighted 30 day mortality was higher in these hospitals, differences in the case mix adjusted odds of 30 day mortality between quarters was less striking, suggesting that factors apart from underuse of guideline recommended treatments were contributing to the excess mortality in these hospitals. The low use of guideline recommended treatment in such hospitals may be the “canary in the coal mine” that signals difficulties in implementing high quality care.^30^ These implementation difficulties might include vulnerability of patients and other hospital level and regional factors,^30^ ^31^ emphasising the importance of looking beyond the prescription of guideline recommended drugs in developing quality improvement programmes. In this scenario, quality improvement might best be achieved not only by targeting underperforming hospitals but also by the development of system-wide initiatives with the aim of delivering equitable management across all national hospitals from time of admission through to discharge and beyond. 

Involving patients in the development and communication of evidence based discharge plans is important and may improve care and outcomes. This no doubt accounted for the success of the UK’s National Service Framework for Coronary Heart Disease in 2000,^34^ but Sweden has a more comprehensive nationwide programme of quality improvement that includes public reporting of hospital mortality, rapid diffusion of new technologies,^35^ more complete use of evidence based practice,^36^ and a more established system for evaluating and reporting the quality and outcomes of care.^37^ This system-wide approach probably accounts, at least in part, for the reduced variation in hospital care of patients with acute myocardial infarction in Sweden. Our results suggest that a national UK strategy aimed at increasing the use of guideline recommended treatments in hospitals in the lowest quarter, combined with system-wide quality improvement for better adherence to guideline recommended treatment, may reduce hospitals’ variation in care to Swedish levels with a leftward shift of the distribution of case mix standardised 30 day hospital mortality in figure 4.

### Limitations of study

Our study has limitations. Firstly, the contribution that other case mix factors unavailable in the data from either or both countries, such as angiography, make to variability in hospital outcomes cannot be determined. These factors at the hospital level may introduce an ecological fallacy when estimating lives saved in hospitals in lower treatment use quarters. However, the problem of additional confounding arises only insofar as factors add prediction beyond the variables already included in the model. Additional sensitivity analyses including treatment variables in the model gave similar results (supplementary figure B). This suggests that study findings based on our approach (using population data from ongoing nationwide clinical registries, measures based on clinical guidelines and professional led views of their importance and comparability, and multilevel modelling adjusted for patient level and hospital level variables) are accurate and robust.

Secondly, we could not evaluate international differences in care for acute myocardial infarction before hospital admission that may potentially influence in-hospital treatment and outcomes. This is unlikely to explain our findings of higher variation in the UK, because we found that time from onset of symptoms to admission, an indicator of pre-hospital management, was similar to that in Sweden.^14^

Thirdly, using the index admission may underestimate the outcome. However, as the aetiology and prognosis of subsequent acute myocardial infarctions are different from the initial episode, we applied the same method to identify the index admission in both countries to reduce the likelihood of including subsequent acute myocardial infarctions.

Fourthly, the national registries do not capture all patients admitted with acute myocardial infarction, and this may be more common in the UK than in Sweden.^38^ Patients who are not captured tend to be older and to have non-ST elevation myocardial infarction.^12^ ^13^ ^38^ We did a sensitivity analysis comparing the case mix in all acute myocardial infarction patients and subgroups of patients aged 80 years and above and patients with non-ST elevation myocardial infarction in Sweden and the UK (supplementary table C). The results showed that differences in case mix comparing all patients and patients with a greater risk of being missed by the registries in the two countries were similar.

Finally, missing values may introduce bias in our case mix model. However, we have previously reported that estimates for the association between case mix variables and 30 day mortality, based on multiple imputed data, were verified by estimates based on complete case analysis.^14^

### Need for further research

International comparisons are an integral component of contemporary research on prognosis.^39^ They are informative about the relative performance of healthcare systems, and further comparisons with other national registries are now needed, including those in the United States,^40^ China,^41^ and New Zealand.^42^ In these countries, participation in registries is often voluntary and research will be needed to understand the effect of hospital and case selection. Each patient with acute myocardial infarction may have had various measurements taken for therapeutic decisions and monitoring. This information is recorded in diverse electronic health record systems before, during, and after hospital admission. Linkage to national electronic health records has been shown to effectively enable studies to investigate and compare fatal and non-fatal events occurring along the care pathway,^43^ including primary care,^44^ hospital care,^14^ ^45^ and post-acute phase care.^46^ ^47^ Increasing availability of linked data on existing health measurements during the care process can facilitate further in-depth and thorough international comparisons for better outcomes for patients. Our findings also have policy implications for identifying, reporting, and thereafter reducing inter-hospital variability in healthcare. Further work is necessary to determine the competing benefits of targeting underperforming hospitals or developing a national programme of quality improvement for more complete use of treatment for acute myocardial infarction across all hospitals.

### Conclusions

Between hospital variation in guideline recommended treatment of acute myocardial infarction was greater in the United Kingdom than in Sweden. This was associated with, and may partly account for, the higher 30 day mortality in UK hospitals and greater variation in that mortality. A system-wide national programme modelled on Sweden’s quality improvement programme has the potential to reduce unacceptable variation in practice if implemented across all UK hospitals, with an anticipated reduction in 30 day mortality towards Swedish levels.

**What is already known on this topic**

Variation between hospitals in quality of care and clinical outcomes provides useful insight into the performance of national health systems
A significant difference in patients’ 30 day mortality after acute myocardial infarction between Sweden and the UK has been reported
International comparisons of between hospital variation and its association with acute myocardial infarction mortality remain absent

**What this study adds**

Hospitals’ 30 day mortality rates for acute myocardial infarction and between hospital variation in mortality were greater in the UK than in Sweden
This was associated with, and may be partly accounted for by, the greater variation in practice as regards guideline recommended treatment for acute myocardial infarction in the UK hospitals
More consistent healthcare across all hospitals with better use of guideline recommended treatment may not only reduce practice variation but also deliver improved clinical outcomes for patients with acute myocardial infarction

## Notes

Ethical approval: The study was approved by the MINAP Academic Group and the Steering Group of SWEDEHEART.

Data sharing: Details of how to obtain additional data from the study (such as technical appendix, statistical programming) are available from the corresponding author at s.chung@ucl.ac.uk.

Transparency declaration: The lead author (the manuscript’s guarantor) affirms that the manuscript is an honest, accurate, and transparent account of the study being reported; that no important aspects of the study have been omitted; and that any discrepancies from the study as planned (and, if relevant, registered) have been explained.

## Notes

Cite this as: *BMJ* 2015;351:h3913
