# Supplementary material for: Comparison of hospital variation in acute myocardial infarction care and outcome between Sweden and United Kingdom: population based cohort study using nationwide clinical registries
Source: BMJ. 2015 Aug 7;351:h3913. doi: 10.1136/bmj.h3913 (PMC4528190; doi:10.1136/bmj.h3913)
Supplement: Supplementary file 1 [file chus024391.ww1_default.pdf]

Supplementary materials

Comparison of hospital variation in acute myocardial infarction care and outcome between Sweden and the United Kingdom

Sheng-Chia Chung, Johan Sundström, Christopher Gale, Stefan James, Lars Wallentin, John Deanfield, Adam Timmis, Tomas Jernberg, and Harry Hemingway

Appendix 1: management of missing values for case mix modelling

The extent of missing data in RIKS-HIA and MINAP are shown in the table. To appropriately utilize all available data, specifically for the case mix model of 30-day mortality in Sweden and the UK, we imputed missing case mix variables using the SAS multiple imputation procedures, replacing each missing value with a set of values which represent a random sample of the missing value.[1] We used the Markov Chain Monte Carlo algorithm for arbitrary missing data patterns.[2] The number of imputations in the study is 21.

In multiple imputation for missing case mix information, we include case mix variables of gender, smoking, history of diabetes and hypertension, troponin, admission systolic blood pressure, admission heart rate, history of cardiovascular disease (heart failure, cardiac arrest prior to admission, cerebrovascular disease, MI), procedure and medication prior to hospital admission (anti-platelet treatment, PCI, CABG), treatment applied for all AMI patients (non-primary PCI, Intravenous glycoprotein IIb/IIIa receptor inhibitors use, heparin use), the follow-up time and vital status of each patient during the entire study period from 2004 to 2010. For analyses based on multiple imputed data, imputed values for categorical variables were not rounded to avoid bias in the estimates.[3 4]

The extent of missingness (%) of case mix variables for multivariate models of the study

Variables of interest	RIKS-HIA N=119,786	MINAP N=391,077
STEMI	2	0
Age, year	0	0
Female	0	0
**MI severity**		
Systolic blood pressure, mmHg	12	14
Heart rate, beat per minute	11	14
Troponin categories	7	29
**Risk Factor**		
Current smoking	10	9
History of diabetes	1	5
History of hypertension	2	6
**Cardiovascular Disease History**		
Heart Failure	6	9
Cardiac arrest prior to admission	0	5
Cerebrovascular disease	19	9
Myocardial infarction	1	6
**Pre-hospital Treatment**		
Antiplatelet therapy	1	5
Prior PCI	1	8
Prior CABG	1	8

Appendix 2: model fit

The model fit was examined by the distribution of Pearson residuals and mean square weighted deviation (Pearson chi-square statistic divided by degree of freedom).[5] Pearson residual plots of the case mix models do not show systematic deviation from normality. A value closer to 1 indicates a good fit. From the 21 imputed datasets, the rang of mean square weighted deviation of case mix models for 30-day mortality is from 1.02 to 1.03 in Sweden and 0.96 to 0.97 in the UK, suggesting a good fit.

References

1. DB. R. *Multiple imputation for nonresponse in surveys.* . New York: Wiley, 1987.
2. Yuan Y. Multiple Imputation for Missing Data: Concepts and New Development. Twenty-Fifth Annual SAS Users Group International Conference. Cary, NC: SAS Institute, 2000.
3. Allison PD. Imputation of categorical variables with PROC MI. . The 30th Annual SAS Users Group International Conference (SUGI 30). Philadelphia, PA;, 2005.
4. Ake CF. Rounding after multiple imputation with non-binary categorical covariates. . The 30th Annual SAS Users Group International Conference (SUGI 30) Philadelphia, PA, 2005. .
5. Brown H, Prescott R. *Applied Mixed Models in Medicine*. West Sussex, England: John Wiley, 2006.

Appendix 3: estimated deaths delayed or deferred

The table summarized the case mix standardised mortality by hospital treatment quartiles, and estimated deaths delayed or prevented if hospitals of the lower treatment use quartiles (Q1-Q3) had hospital case mix-standardized mortality in the highest treatment quartile (Q4). The greatest mean difference in risk-standardised mortality observed between the lowest and highest hospital use quartiles was primary PCI for STEMI in Sweden (2.4%), any reperfusion and discharge dual antiplatelet in the UK (0.9%). Compared to hospital reperfusion therapy use quartiles, difference in case mix standardised mortality between the highest and lowest hospital use quartiles was smaller for most discharge medications.

In absolute number of death prevented or deferred, decreasing the case mix standardised mortality discrepancy between hospital primary PCI use quartiles resulted an estimated 581 lives saved in Sweden, and 1013 lives saved in the UK. Deaths prevented or deferred from decreasing mortality difference between hospital discharge mediation use quartiles was estimated to be 573 in Sweden and 2274 in the UK for dual antiplatelet, 574 for statin in Sweden, and 1579 for beta blocker In the UK. The similar or greater estimated number of lives saved by hospital practise variation in discharge medications use than reperfusion was due to the greater patients population (all AMI survived the acute infarct) may be benefited from the use of discharge meditation than reperfusion therapy for STEMI patients alone.

Estimated deaths delayed or prevented if hospitals of the lower treatment use quartiles (Q1-Q3) had hospital standardised mix-standardised mortality in the highest treatment quartile (Q4).

Hospital treatment quartiles	Sweden			UK		
	Standardised Mortality[1]	N of patients	Estimated death prevented	Standardised Mortality[1]	N of patients	Estimated death prevented
Primary PCI for STEMI						
Lowest	8.8	11722	281	11	113406	794
Medium-low	8.2	9038	163	10.4	14607	15
Medium-high	8	8565	137	11.8	13633	204
highest	6.4	9107	0	10.3	15663	0
Sum			581			1013
Any reperfusion for STEMI						
Lowest	8.5	9567	96	11.2	39208	353
Medium-low	8.1	9893	59	11.3	39411	394
Medium-high	8.1	9659	58	10.9	39266	236
highest	7.5	9313	0	10.3	39452	0
Sum			213			983

Revascularization for NSTEMI						
Lowest	7.6	20279	183	10.1	50296	402
Medium-low	7.4	20655	145	10.3	64986	650
Medium-high	6.8	19580	20	9.5	58492	117
highest	6.7	20840	0	9.3	58513	0
Sum			348			1169
Anticoagulant for NSTEMI						
Lowest	7.3	20593	0	9.4	60046	-180
Medium-low	7.3	20195	0	10.3	41490	249
Medium-high	6.8	20066	-100	9.8	52658	53
highest	7.3	20499	0	9.7	67430	0
Sum			-100			122
Any antiplatelet						
Lowest	7.6	30249	181	10.1	100023	500
Medium-low	7.8	30314	243	10	98973	396
Medium-high	7.5	29865	149	9.9	96779	290
highest	7	29356	0	9.6	95270	0
Sum			573			1186
Dual antiplatelet						
Lowest	7.7	30408	213	10.2	100320	903
Medium-low	7.6	30357	182	10.1	98888	791
Medium-high	7.6	29610	178	9.9	96584	580
highest	7	29409	0	9.3	95253	0
Sum			573			2274
Beta-blocker						
Lowest	7.3	29323	0	10.1	100274	602
Medium-low	7.8	30298	151	10	98894	494
Medium-high	7.6	29946	90	10	96615	483
highest	7.3	30217	0	9.5	95238	0
Sum			241			1579
ACEI/ARB						
Lowest	7.6	30194	91	10	99432	199
Medium-low	7.5	29829	60	10	98008	196
Medium-high	7.4	29893	30	9.8	97657	0
highest	7.3	29868	0	9.8	95910	0
Sum			181			395
Statin						
Lowest	7.7	30772	215	10.3	100900	605
Medium-low	7.4	29566	118	10.1	97867	391

Medium-high	7.8	30142	241	9.6	96483	-96
highest	7	29304	0	9.7	95644	0
Sum			574			900

[1]Hospital mortality standardised by patient case mix of the country.

Supplementary tables and figures

Table A: Median and interquartile range of hospital-specific case mix proportions and mean values by admission year in Sweden and the UK *

Patient characteristics at hospital level

	Sweden			UK		
Year	2004	2007	2010	2004	2007	2010
Number of hospitals providing care for AMI	75	76	81	233	228	225
STEMI	31.2, 8	27.8, 12.3	31.3, 11.2	38, 23.8	40.2, 25.9	27.5, 30.5
Age, years	72.2, 3.1	71.2, 3.2	70.7, 2.2	69.4, 3.7	69.2, 4.4	69.9, 4.4
Female	37.6, 6.7	36.9, 6	35.1, 7.1	34.3, 7.9	33.7, 7.3	34.7, 8.6
MI severity						
Systolic blood pressure, mmHg	144.3, 10	145, 10	145, 8.5	140, 7.5	139, 7	139, 7
Heart rate, beat per minute	77, 5	77, 5	78, 4	78.3, 4	79.5, 3.5	79, 4
Troponin I, ng/mL	4.4, 6.4	3.7, 5	3.1, 3.4	7, 16	4.3, 7.8	2.3, 3.4
Troponin T, ng/mL,	1, 2.7	0.7, 0.6	0.8, 1.6	0.9, 2	0.7, 1.4	0.8, 3.5
Risk factor						
Current smoking	22.2, 6.3	22.4, 6.1	23.1, 6.6	30.8, 9	29.5, 10.9	26.6, 10.4
History of diabetes	23.7, 4.4	22.2, 5.9	22.3, 5.7	16.1, 5.9	16.7, 6.2	19.4, 5.8
History of hypertension	42.4, 8.9	46.5, 9.1	49.1, 7.6	43.5, 9.4	47.8, 11.8	49.7, 10.7
History of CVD						
Heart failure	12.5, 6.2	9.3, 6.7	6.7, 4.1	5.2, 5.1	4, 4.6	4.7, 4.8
Previous MI	29.4, 6	21.5, 5.1	18.7, 4.1	20, 6.5	17.1, 6.3	17.3, 6.7
Cardiac arrest	0.9, 1.6	1.2, 1.2	1.4, 1.9	1.8, 2.1	1.8, 1.7	1.9, 2.6
Stroke	7, 14.3	10, 4.3	8.6, 3.7	7.6, 5.4	7.5, 5.1	8.9, 5.1
Treatment prior to admission						
Single antiplatelet	41.1, 7.4	37, 6.8	33.5, 7.1	20.1, 19.6	26.9, 15	22.1, 19.5
Combined antiplatelet	3, 3	3.8, 2.5	3.3, 2.7	0, 0	2.7, 3.8	2.1, 3.4
PCI	6.8, 3	7.6, 4.4	8.4, 3.6	3.4, 4.4	5.6, 4.7	6.1, 4.7
CABG	7.2, 3.3	7.8, 3.3	7.4, 3.2	3.9, 2.5	4.4, 3.1	5.4, 3.1
Volume	2325, 1680	2325, 1739	2349, 2845	1982, 1435	2102, 1372	2084, 1589

* Distribution of hospital-specific % for STEMI, female, all risk factors, history of CVD, treatment prior to admission; distribution of hospital-specific mean for age; distribution of hospital-specific median for systolic blood pressure, heart rate, and troponin values. Median and interquartile range of hospital proportions.

Table B: Correlation between case mix standardised hospital proportions of treatment use and death at 30-day in Sweden and the UK

	Sweden (87 hospitals)		UK (242 hospitals)	
	Correlation coefficient	% of mortality variation explained	Correlation coefficient	% of mortality variation explained
Hospital STEMI treatment				
Primary PCI	-0.3654	13.35	-0.247	6.12
Fibrinolysis[1]	0.2768	7.66	0.112	1.25
Hospital NSTEMI treatment				
Revascularization	-0.2382	5.67	-0.273	7.47
Anticoagulant	0.2487	6.19	0.09	0.82
Hospital discharge medication for all AMI				
Any antiplatelet	-0.2785	7.75	-0.345	11.88
Dual antiplatelet	-0.0125	0.02	-0.278	7.72
Beta-blocker	0.0989	0.98	0.003	0
ACEI/ARB	-0.1414	2.00	-0.134	1.8
Statin	-0.3606	13.00	-0.353	12.47
Overall		28.1		21.6

[1]partial correlation between case mix adjusted hospital fibrinolysis rate and 30-day mortality, accounting for hospital primary PCI rate.

Figure A: Case mix-adjusted odds ratio for AMI 30-day mortality by year and hospital reperfusion quartiles in Sweden and the UK.

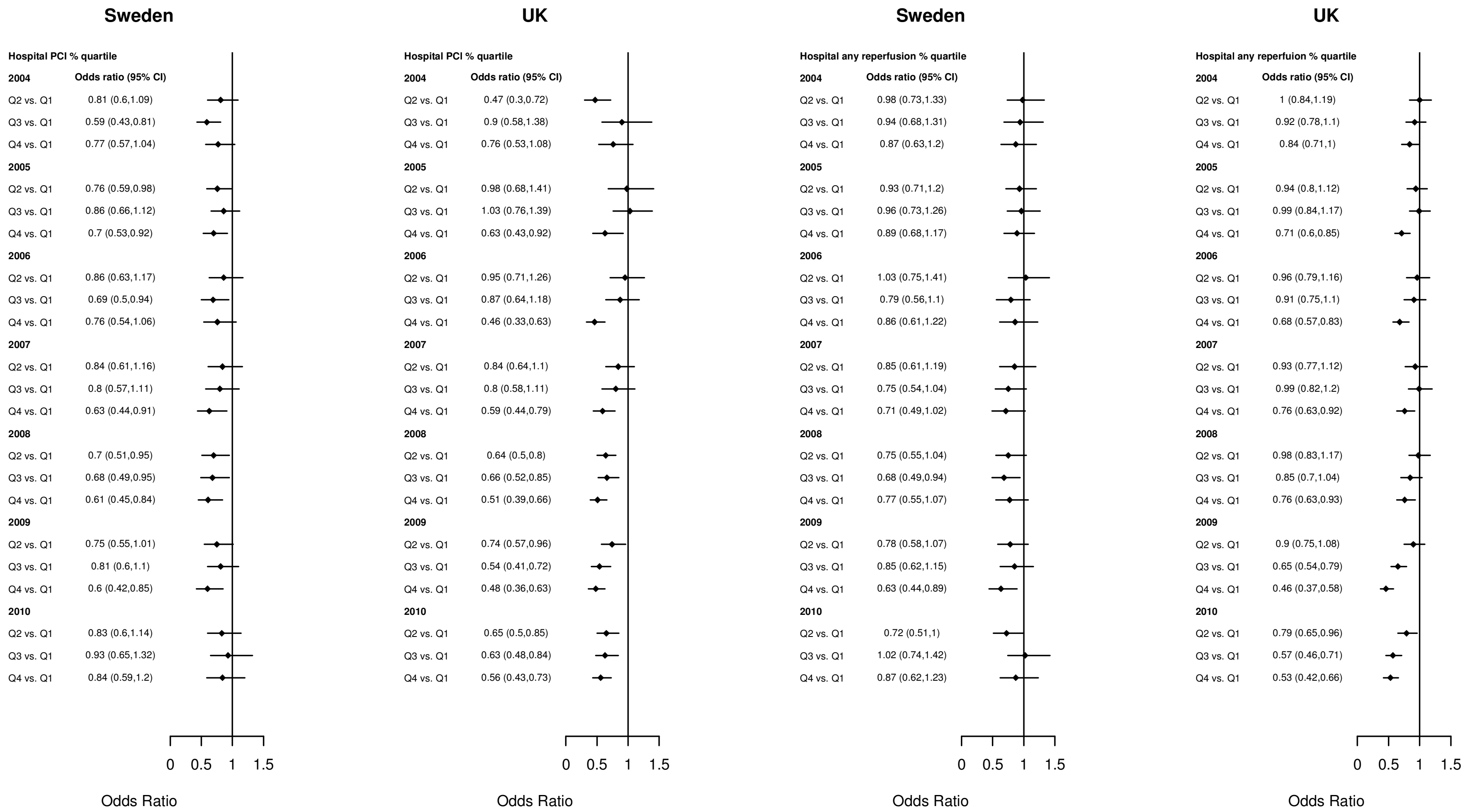

Figure B: Hospital variation in case mix and treatment standardized 30-day mortality (%) in Sweden and the UK, 2004-2010.

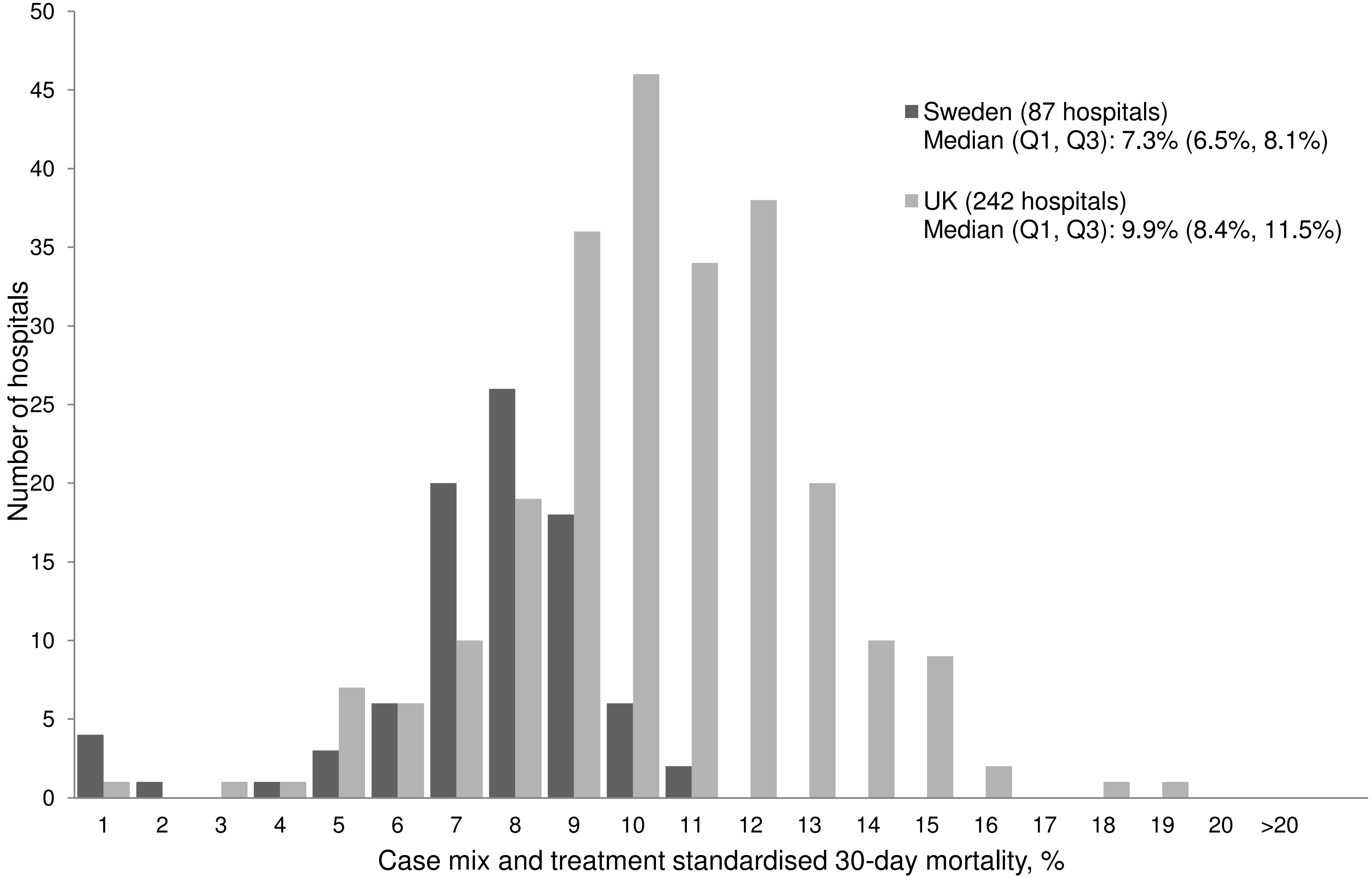

Table C: Case-mix and treatment for patients with acute MI in Sweden (n=119,786) and the UK (n=391,077), comparing all patients to patient subgroups (age ≥ 80 years old, NSTEMI). Values are % (95% confidence interval) unless otherwise indicated

	Sweden			UK		
**Case-mix**<br>**N**	**All AMI**<br>**119786**	**Age ≥ 80 years**<br>**35903**	**NSTEMI**<br>**81354**	**All AMI**<br>**391077**	**Age ≥ 80 years**<br>**101711**	**NSTEMI**<br>**233659**
STEMI	32.1 (31.8, 32.3)	24.9 (24.5, 25.3)	--	40.3 (40.1,40.4)	27.3 (27, 27.6)	--
Age, year, mean, SD	71.2, 12.3	85.0, 3.8	72.4, 12.0	69.5, 13.6	85.8, 4.3	71.8, 13.3
Female	36.3 (36.1, 36.6)	51 (50.5, 51.5)	37.6 (37.3, 37.9)	34.8 (34.7,34.9)	53.4 (53, 53.7)	38 (37.8, 38.2)
**MI severity, median (Q1, Q3)**						
Systolic blood pressure, mmHg	145 (125, 165)	140 (120, 163)	148 (130, 167)	139 (120, 158)	137 (117, 158)	140 (121, 160)
Heart rate, beat per minute	78 (65, 93)	80 (68, 100)	79 (66, 95)	79 (66, 94)	83 (70, 100)	80 (68, 97)
Troponin I, µg/L	4.2 (0.8, 18.0)	4.3 (0.94, 17.1)	2.6 (0.63, 9.69)	4.4 (0.8, 21.7)	3.52 (0.68, 15.9)	2.18 (0.46, 8.36)
Troponin T, µg/L	0.7 (0.2, 2.3)	0.7 (0.21, 2.2)	0.45 (0.17, 1.3)	0.65 (0.2, 2.3)	0.53 (0.176, 1.82)	0.356 (0.14, 0.96)
**Risk factor**						
Current smoking	23.3 (23.0,23.5)	6.7 (6.4, 7)	19.9 (19.6, 20.2)	29.5 (29.3,29.6)	8 (7.8, 8.2)	23.5 (23.3, 23.7)
History of diabetes	22.7 (22.4,22.9)	22.4 (21.9, 22.8)	24.7 (24.4, 25)	17.6 (17.4,17.7)	17.4 (17.2, 17.7)	20.7 (20.5, 20.8)
History of hypertension	45.2 (44.9,45.5)	50.6 (50.1, 51.1)	47.8 (47.4, 48.1)	47.3 (47.2,47.5)	54.4 (54.1, 54.7)	51 (50.8, 51.2)
**Cardiovascular disease history**						
Heart Failure	9.7 (9.5,9.8)	18.3 (17.9, 18.8)	12.3 (12.1, 12.5)	5.3 (5.2,5.4)	10.9 (10.7, 11.1)	7.4 (7.3, 7.5)
Cardiac arrest prior to admission	1.3 (1.3,1.4)	0.8 (0.7, 0.9)	0.8 (0.8, 0.9)	2.3 (2.2,2.3)	1.3 (1.3, 1.4)	1.3 (1.2, 1.3)
Cerebrovascular disease	10.1 (9.9,10.3)	16.1 (15.7, 16.5)	11.4 (11.1, 11.6)	8.5 (8.4,8.5)	14.4 (14.2, 14.7)	10.2 (10.1, 10.3)
MI	22.4 (22.1,22.6)	31.9 (31.4, 32.4)	26.5 (26.2, 26.8)	18.3 (18.1,18.4)	24 (23.8, 24.3)	22.9 (22.7, 23.1)
**Pre-hospital treatment**						
Antiplatelet mono-therapy	36.6 (36.3,36.6)	51.7 (51.1, 52.2)	42 (41.7, 42.4)	26.4 (26.3,26.6)	33.5 (33.2, 33.8)	31.2 (31, 31.4)
Antiplatelet dual-therapy	4.0 (3.9,4.1)	4.4 (4.2, 4.6)	4.8 (4.7, 5)	2.9 (2.9,3)	3.4 (3.3, 3.5)	3.7 (3.6, 3.8)
PCI	8.0 (7.8,8.2)	5.7 (5.4, 5.9)	9.2 (9, 9.4)	5.4 (5.4,5.5)	3.1 (3, 3.2)	6.3 (6.2, 6.4)
CABG	7.7 (7.6,7.9)	7.8 (7.6, 8.1)	9.8 (9.6, 10)	4.8 (4.8,4.9)	4.1 (3.9, 4.2)	6.5 (6.4, 6.6)

Abbreviations: STEMI, ST-elevation myocardial infarction; MI, myocardial infarction; PCI, percutaneous coronary intervention; CABG, coronary artery bypass graft surgery;
